# Keap1 recognizes EIAV early accessory protein Rev to promote antiviral defense

**DOI:** 10.1371/journal.ppat.1009986

**Published:** 2022-02-09

**Authors:** Yan Wang, Guanqin Ma, Xue-Feng Wang, Lei Na, Xing Guo, Jiaqi Zhang, Cong Liu, Cheng Du, Ting Qi, Yuezhi Lin, Xiaojun Wang

**Affiliations:** State Key Laboratory of Veterinary Biotechnology, Harbin Veterinary Research Institute of Chinese Academy of Agricultural Sciences, Harbin, China; Duke University Medical Center, UNITED STATES

## Abstract

The Nrf2/Keap1 axis plays a complex role in viral susceptibility, virus-associated inflammation and immune regulation in host cells. However, whether or how the Nrf2/Keap1 axis is involved in the interactions between equine lentiviruses and their hosts remains unclear. Here, we demonstrate that the Nrf2/Keap1 axis was activated during EIAV infection. Mechanistically, EIAV-Rev competitively binds to Keap1 and releases Nrf2 from Keap1-mediated repression, leading to the accumulation of Nrf2 in the nucleus and promoting Nrf2 responsive genes transcription. Subsequently, we demonstrated that the Nrf2/Keap1 axis represses EIAV replication via two independent molecular mechanisms: directly increasing antioxidant enzymes to promote effective cellular resistance against EIAV infection, and repression of Rev-mediated RNA transport through direct interaction between Keap1 and Rev. Together, these data suggest that activation of the Nrf2/Keap1 axis mediates a passive defensive response to combat EIAV infection. The Nrf2/Keap1 axis could be a potential target for developing strategies for combating EIAV infection.

## Author summary

Here we report that the Nrf2/Keap1 axis acts as an antiviral effector on EIAV replication through two-way regulation of Rev. On the one hand, EIAV-Rev activates the Nrf2/Keap1 axis through a competitive interaction with Keap1, and promotes the transcription of cytoprotective genes implicated in the antiviral response. On the other hand, Keap1 restricts Rev/RRE-dependent RNA transport, leading to inhibition of viral protein synthesis and a reduction in viral replication. This study highlights the importance of the Nrf2/Keap1 axis in controlling EIAV replication, and identifies this pathway as a potential new entry-point for treating lentivirus infection. Importantly, this report provides new insights into the regulation of viral replication by the Nrf2/Keap1 axis.

## Introduction

Equine infectious anemia virus (EIAV), an equine lentivirus, causes persistent infection characterized by recurring febrile episodes associated with EIA clinical signs [1,2]. Unlike hosts with infections caused by other lentiviruses, most horses infected with EIAV become lifelong inapparent EIAV carriers by eliciting immune control over virus replication [3–5]. An attenuated EIAV vaccine was developed through long-term passaging in equine macrophages *in vitro* and has successfully controlled the EIA epidemic in China, indicating that protective immunity can be induced after virus infection [5–7]. EIAV has therefore been widely accepted as a system for identifying potential immune control over lentiviruses [1,8,9]. Previous studies have shown that the initial innate immune response blocks EIAV replication and that the virus has some mechanisms to counteract innate immunity restriction [2,10–13]. However, the factors that determine whether infection causes death of the host or whether the host progresses to an inapparent carrier remain unknown [1,2,9]. Elucidation of the innate cellular defenses during EIAV infection could facilitate comprehension of the interplay between immune control and EIAV infection.

Oxidative stress is commonly induced by viral invasion, and plays a critical role in the pathogenesis of various viruses [14–17]. The activation of the antiviral and inflammatory signaling pathways has also been linked with the production of ROS [15,17–19]. Simultaneously, the nuclear factor erythroid 2-related factor 2 (Nrf2) antioxidant pathway, which is induced downstream by ROS, can be activated to maintain cellular redox homeostasis by regulating antioxidant genes and phase II detoxification enzymes [20–22]. Moreover, several studies have demonstrated that the balance of redox homeostasis contributes to viral pathogenesis through multiple mechanisms [23–25]. The interaction of Nrf2 with its cellular inhibitor, Keap1, comprises a conserved and important intracellular antioxidant defense system [26,27]. Under normal physiological conditions, Nrf2 is sequestered in the cytoplasm by its regulatory protein, Keap1. However, under oxidative stress, the interaction between Nrf2 and Keap1 is disrupted and the activated Nrf2 is sequentially translocated to the nucleus, where it binds the antioxidant response elements (AREs) and inducing expression of several antioxidant genes [27,28]. As well as being involved in the anti-oxidative response, Nrf2 is also an important immune-modulator, and interferes with pro-inflammatory genes as well as innate immune signals such as TLRs, NF-ҡB, and metastasis [21,23,25,28]. A growing body of evidence from studies of infection from various different viruses links the Nrf2 signal to virus replication [16,29–32].

Recently, it has been reported that ROS and inflammatory cytokines (IL-1β and TNF-α) were generated following EIAV infection [33,34]. Moreover, oxidative stress-induced damage and alterations in redox status have been associated with increasing disease severity in EIAV-infected horses [4]. Importantly, a transcriptomics screen as part of our previous study found that the Nrf2/Keap1 axis was activated during EIAV infection. These observations indicate a possible role for redox homeostasis in combating EIAV infection. However, whether or how EIAV infection activates the Nrf2 pathway, and the potential role of this pathway in antagonizing EIAV replication remain unclear. In the current study, we found that EIAV infection activates the Nrf2/Keap1 defense system through the EIAV accessory protein Rev. Mechanistically, Rev binds directly to the Kelch domain of Keap1, and suppresses its function, which is to inhibit Nrf2. This liberates Nrf2 and triggers the Nrf2 pathway. Moreover, the activation of Nrf2/Keap1 axis inhibits EIAV replication. We further demonstrate that the Nrf2/Keap1 axis is able to inhibit EIAV replication via two independent mechanisms: (i) enhancing antioxidant gene transcription and (ii) the repression of Rev-mediated RNA transport, leading to reduce viral protein synthesis. In general, EIAV Rev-triggered activation of Nrf2/Keap1 axis following EIAV infection contributes to the host defenses against infection.

## Results

### EIAV Infection induces the antioxidant response and up-regulates total Nrf2 and phospho-Nrf2

**Fig 1:**
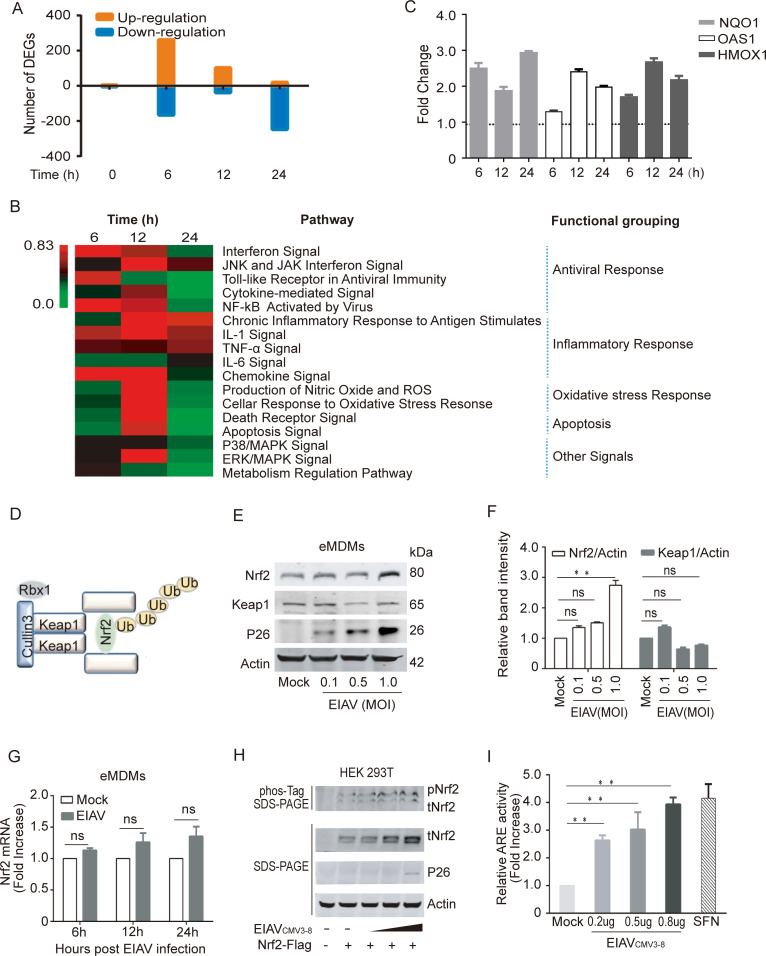
The Nrf2-Keap1 axis is activated by EIAV infection. **(A)** Waterfall plot representing the total number of up- and down-regulated genes at each time point following transcriptome analysis (DEGs, differentially expressed genes; selected based on fold change>1.5, P value<0.05). **(B)** Heat map showing statistically significant canonical pathways commonly regulated at 6 h, 12 h and 24 h post EIAV infection, compared to the control. Heat map colors represent the ratio of regulated genes after EIAV infection (red and green correspond to over- and under-regulated genes, respectively). **(C)** Real-time PCR analysis of NQO1, OAS1 and HMOX1 mRNA in equine macrophages infected with EIAV at the indicated times (6 h, 12 h and 24 h) post infection. **(D)** Schematic representation of Nrf2-Keap1 interaction. **(E)** Quantification of Nrf2 and Keap1 expression in equine macrophages infected with EIAV at varied infection dose. **(F)** Densitometric analyses of pNrf2 and tNrf2 band intensity shown after normalization to actin. **(G)** Nrf2 mRNA was quantified using real-time PCR as above. **(H)** The effects of EIAV infection on the phosphorylation of Nrf2 were analyzed using a Phos-tag assay. Immunoblots for total Nrf2, p26 and actin were performed on normal SDS-PAGE gels as previously described. **(I)** The activation of Nrf2/Keap1 axis triggered by EIAV infection was analyzed using the ARE reporter gene assay. 293T cells were co-transfected together with the ARE luciferase reporter plasmid, as well as pcDNA3.1 (empty vector) or increasing concentrations of EIAV_CMV3-8_. Twenty-four hours later, cells were lysed and firefly luciferase activities was assayed. Data are representative of two (A-B) or three (C-I) independent experiments.

To investigate the cellular response to EIAV infection, we performed a transcriptome analysis of EIAV infected equine macrophages (eMDMs). Based on the obtained transcriptomic data, the observed changes in gene expression following EIAV infection were first represented using a waterfall plot, as shown in Fig 1A. More than 400 genes were either up- or down-regulated within 6 h of viral infection compared with the sham control, which indicated that most changes in gene expression occur at the early stages of infection. The subsequent analysis of intracellular signaling pathways showed that multiple canonical pathways were regulated coordinately by EIAV infection. In particular, the antiviral pathways and the inflammatory pathways were highly enriched following EIAV infection (Fig 1B). Moreover, 12 h following initial viral infection of cells, an enrichment in the networks associated with the oxidant stress relative response was observed (Fig 1B). Nrf2 responsive genes, including NQO1, OAS1 and HMOX1, were also up-regulated at this time (Fig 1B and 1C), indicating the involvement of the Nrf2 pathway in the EIAV infection-mediated intracellular response. To further verify this observation, we analyzed the levels of Nrf2 in the EIAV-infected macrophages separately at both the RNA and protein levels, using real-time PCR and western blotting. The results showed that EIAV infection did indeed up-regulate levels of Nrf2 protein in a dose-dependent manner (Fig 1E and 1F), but not levels of mRNA (Fig 1G). In addition, we found that levels of total Nrf2 (tNrf2) and phosphorylated Nrf2 (pNrf2) in 293T cells transfected with an EIAV infectious clone were also elevated in a dose-dependent manner (Fig 1H).

To further assess the impact of EIAV infection on the Nrf2 pathway, an ARE promotor-based luciferase reporter system was developed for the genes downstream of Nrf2. An EIAV infectious clone and the ARE-promoter reporter system were co-transfected into 293T cells. As expected, we observed more than 4-fold elevation in ARE reporter activation by the EIAV clone compared to the mock-transfected control (Fig 1I). Collectively, these results demonstrate that the Nrf2 pathway is activated in EIAV-infected host cells.

### EIAV-Rev activates the Nrf2/Keap1 axis

**Fig 2:**
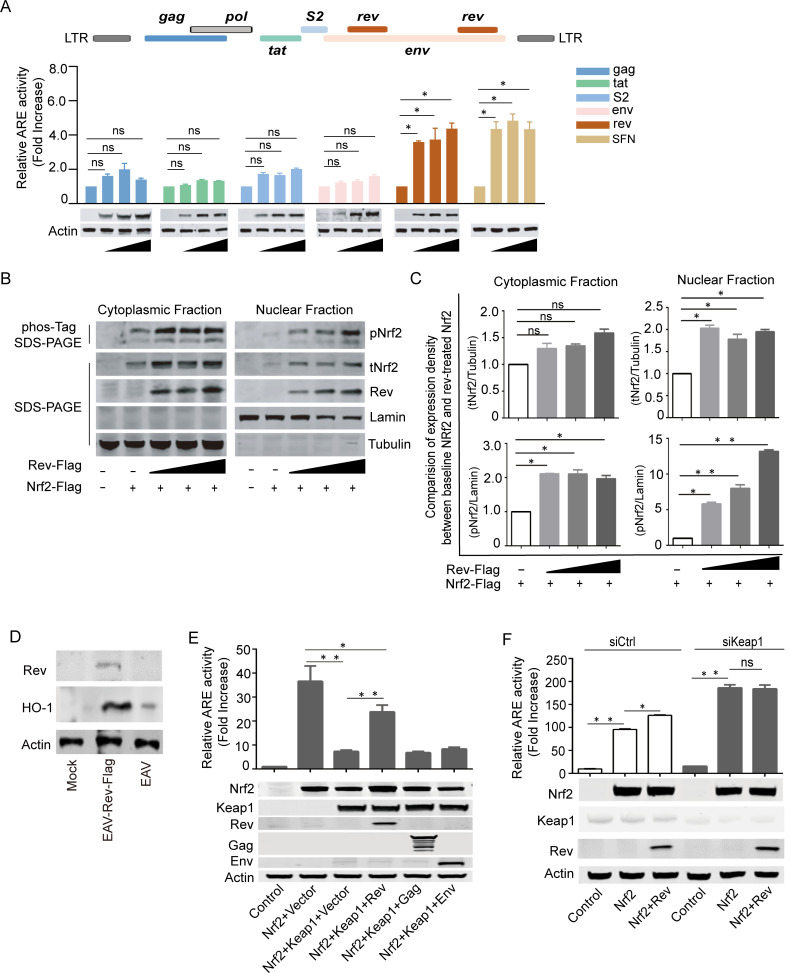
EIAV-Rev Induces Nrf2/Keap1 axis Activation. **(A)** The ability of EIAV-coded proteins inducing Nrf2/Keap1 axis activation were evaluated using the ARE gene reporter. The assay protocol was the same as that shown in Fig 1I but cells were transfected with EIAV-*env*, EIAV-*gag*, EIAV-*rev*, EIAV-*S2* or EIAV-*Tat* separately. SFN was chosen as a positive control. Western blot depicting the expression of each of the transfected constructs, including the loading control, actin. **(B-C)** Rev triggered Nrf2/Keap1 axis activation. 293T cells were transfected with Flag-tagged-*rev*, and the cytoplasmic and nuclear proteins were fractionated and then immunoblotted for pNrf2 and tNrf2. Densitometric analyses of pNrf2 and tNrf2 band intensity shown after normalization to Tubulin (cytoplasmic purity control) or Lamin (nuclear purity control). **(D)** Immunoblot analysis for HO-1 expression of extracts from equine macrophage cells infected with either the EAV infectious clone or EAV-Rev-Flag. **(E)** The ARE gene reporter was assayed for Nrf2 activation in the presence of Rev. 293T cells were transfected with the indicated plasmids. After 24 h, the luciferase activities were assessed (upper) and exogenous expression of proteins was measured using western blotting (lower). **(F)** Same assay protocol as D but 293T cells were pre-treated with siCtrl or siKeap1 and then transfected with the indicated plasmids. Data shown represent three independent experiments. Data are the mean values ± SDs, ns (non-significant), P > 0.05; *P < 0.05; **P < 0.01 (Student’s t test).

To determine which, if any, EIAV-coded proteins are crucial for Nrf2 activation, different amounts of expression plasmids carrying the EIAV *gag*, *tat*, *s2*, *env*, or *rev* genes were co-transfected into 293T cells together with the luciferase reporter system. We found that expression of the EIAV-Rev protein, but not the other viral proteins, displayed a dose-dependent correlation with the ability to activate the Nrf2/Keap1 axis as strongly as sulforaphane (SFN) (Fig 2A). This suggested an important role of EIAV-Rev in activating the Nrf2/Keap1 axis. To further verify Rev-triggered activation of the Nrf2/Keap1 axis, total Nrf2 (tNrf2) and phosphorylated Nrf2 (pNrf2) levels in the nucleus and cytoplasm of transfected-293T cells with or without Rev were assessed separately. The expression of tNrf2 and pNrf2 increased with the presence of Rev in a dose-dependent manner both in the cytoplasm and nucleus (Fig 2B and 2C), indicating that Rev can stabilize Nrf2 and increase phosphorylation and nuclear translocation of Nrf2.

To confirm direct Nrf2 activation by Rev, an Equine Arteritis Virus (EAV) infectious clone with Rev-Flag was generated following Qi, 2017. This system allowed us to introduce Rev into equine macrophages, while overcoming the difficulties in transfecting primary cells. We found that Rev expression induced the upregulation of HO-1 (a typical Nrf2-inducible gene) in equine macrophages (Fig 2D). These data indicated that Rev can directly trigger Nrf2 activation. Additionally, suppression of Nrf2 by Keap1 was released by Rev, but not Gag or Env, resulting in the recovery of Nrf2 activation (Fig 2E). However, specific siRNA-mediated suppression of Keap1 expression promoted ARE activity to the same level with or without Rev expression when Nrf2 presented, indicating that Keap1 is the key factor in Rev-mediated ARE activation (Fig 2F). Taken together, these lines of biochemical evidence indicate that EIAV-Rev is able to initiate and retain activation of the Nrf2/Keap1 axis with a Keap1-dependent mechanism.

### EIAV-Rev interacts with Keap1 through its Kelch domain

**Fig 3:**
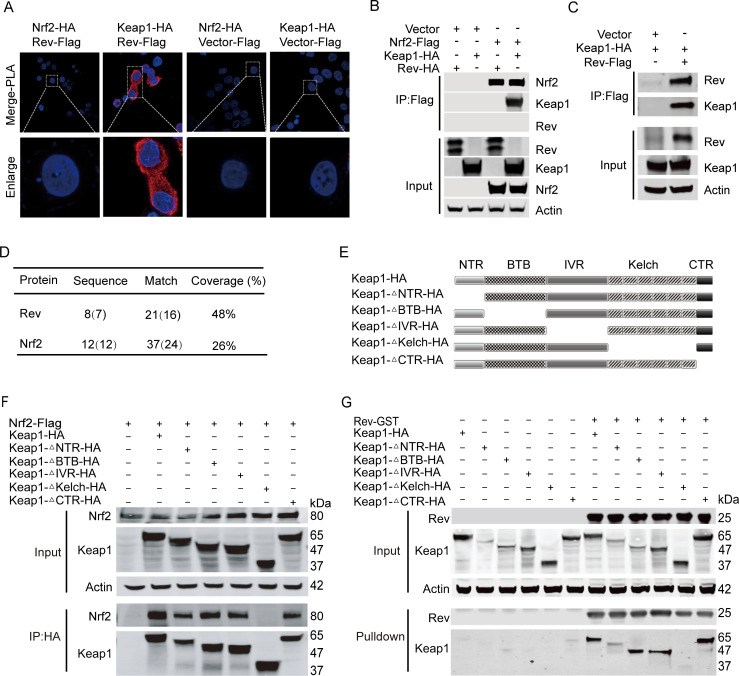
EIAV-Rev interacts with Keap1 but not Nrf2. **(A)** PLA assay was used to analyze the interactions between Rev and Keap1 or Nrf2. The red spots represent interacting complexes of the examined proteins. The nuclei were stained with DAPI (blue). Cells co-transfected with Keap1 (Nrf2) and vector were used as negative controls. **(B)** and **(C)** Reciprocal immunoprecipitations of Rev and Keap1 or Nrf2 were performed on 293T cells co-transfected with plasmids for Nrf2-Flag and *rev*-HA, Nrf2-Flag and Keap1-HA or with Keap1-HA and Flag-*rev*. 24 hours post-transfection, cells were collected and subjected to pull down with Flag beads. **(D)** Mass spectrometry analysis of Rev and Nrf2 peptides after Keap1 pull-down. **(E)** Schematic diagram of Keap1 domain structure and Keap1 deletion mutants used in (E and F). All Keap1 deletion mutants were HA tagged at the amino terminal end. **(F)** The interactions between Keap1 mutants and Nrf2 were screened with Co-IP with the selected antibodies. **(G)** as **(F)** with the addition of GST-tagged-*rev* instead of Nrf2-Flag.

To investigate how Rev induces the activation of the Nrf2/Keap1 axis, we first determined whether there was a physical interaction between Keap1, Nrf2 and Rev using a proximity ligation assay (PLA) and co-immunoprecipitation (Co-IP) assays in 293T. The results showed that Rev has a direct interaction with Keap1 (HA-tagged) but not Nrf2 (Flag-tagged) (Fig 3A–3C). Consistent with this result, an interaction between endogenous Keap1 and Rev was evident in equine macrophages through mass spectrometry analysis of the proteins pulled down by Keap1, and compared with Nrf2, Rev showed a higher peptide coverage (Fig 3D). Next, the crucial domain on Keap1 necessary for the interaction with Rev was identified using a Co-IP assay, with co-transfection of Rev together with various Keap1 mutants, including N-terminal region (NTR), Bric-a-Bric domain (BTB), intervening region (IVR), Kelch domain, and C-terminal domain (CTR) deletions (Fig 3E). Co-IP results showed that Keap1 mutants lacking NTR, BTB, IVR or CTR were precipitated with Rev similarly to the full length Keap1 (Fig 3G). However, the Keap1 mutant without a Kelch domain completely lost the ability to bind Rev (Fig 3G), suggesting that the Kelch domain is required for the interaction with EIAV-Rev. Similarly, this Keap1 mutant (Kelch domain deletion) did not co-precipitate with Nrf2 (Fig 3F), consistent with previous studies [30], indicating that Nrf2 and Rev interact with Keap1 through a similar region (Fig 3F and 3G).

Previous work has suggested that the “DLG” and “ETGE” motifs located in the Nrf2 Neh2 region were required for the binding of Nrf2 and Keap1. By screening Rev amino acid sequences, we also found a “GE” motif (S1A Fig). We therefore sought to discover whether Keap1 interacts with Rev in a similar manner as with Nrf2. To this end, Rev mutants were developed as shown in S1A Fig, defective in either the “DYG” or “RWGE” motifs singly, or defective in both. We then co-expressed Flag-tagged Rev mutants together with HA-tagged Keap1 in 293T cells and assessed the binding between these two proteins using Flag antibody-based Co-IP. Interestingly, these Rev mutants all still possessed the capacity to interact with Keap1 (S1B Fig). These data suggested that there might be another specific sequence on Rev with the ability to bind to Keap1. To gain further insights into the domain(s) of Rev responsible for the interaction with Keap1, we designed three segmental Rev mutants with different area deletions (S1C Fig). Co-IP assays showed that an N-terminal (1-56aa) deletion on Rev led to failure of the interaction between Rev and Keap1 (S1D Fig). Moreover, this N-terminal deletion also resulted in Rev losing the ability to activate the Nrf2/Keap1 axis (S1E Fig). Collectively, these data indicated that 1-56aa at the Rev N-terminal is the key region both for binding Keap1 and activating the Nrf2/Keap1 axis.

### Competitive binding of Nrf2 or Rev to Keap1 in 293T cells

**Fig 4:**
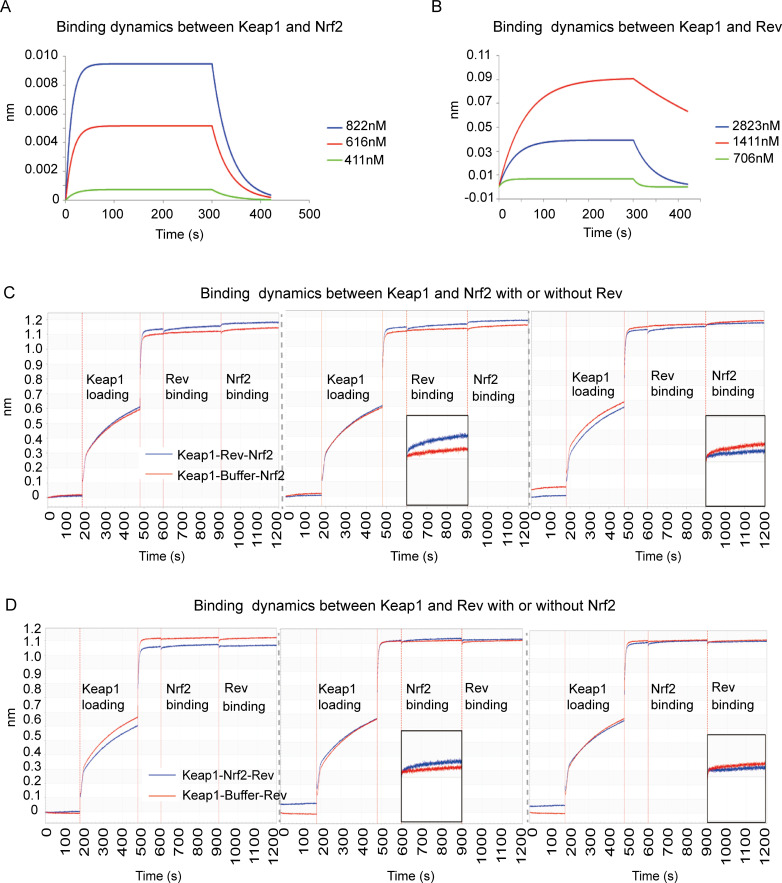
Rev-competitive inhibition antagonizes Nrf2 binding to Keap1. **(A-B)** Biolayer interferometry graphs showing association and dissociation steps using different concentrations of Nrf2 (A) or Rev (B) to Keap1 immobilized on the anti-streptavidin biosensors. **(C-D)** BLI profiles showing the effect of Rev on the affinity of the Keap1-Nrf2 interaction. Rev incubated with Keap1 pre-coated with Nrf2 on the biosensors (C). Nrf2 (blue line) incubated with Keap1 pre-coated with Rev (D). The data are expressed as means and SD for at least three independent replicates.

If Nrf2 and Rev both interact with the Keap1 Kelch domain, it is possible that the binding of Nrf2 and Rev to Keap1 is competitive. To address this question, we performed a biolayer interferometry (ForteBio) assay to test affinity between Keap1, Nrf2 and Rev. The results showed binding between Keap1 and Rev and between Keap1 and Nrf2, regardless of which protein was tethered to the sensor. This biophysical binding result was consistent with the Co-IP results. The binding curves of Keap1-Nrf2 and Keap1-Rev were found to be very similar and with comparable biophysical affinity (KDM Keap1-Nrf2 = 3.995E^-07^ vs KDM Keap1-Rev = 4.9375E^-07^) (Fig 4A and 4B). The binding between Keap1 and Nrf2 in either the presence or absence of Rev was then examined, to determine the competitive effect of Rev to Nrf2 when binding to Keap1. The results showed that it was difficult for Keap1 to interact with Nrf2 (no increasing binding signal was observed for the blue line) when Keap1 and Rev were pre-combined (saturated state), while a significant binding signal was observed in the control group (Keap1-buffer-Nrf2) without pre-binding of Rev and Keap1 (Fig 4C and 4D). Similar binding dynamics were observed when the binding of Keap1 and Rev with or without pre-combination of Keap1 and Nrf2 was tested. Taken together, these results confirmed that Rev and Nrf2 bind competitively to Keap1 *in vitro*.

### EIAV-Rev prevents Keap1-mediated Nrf2 degradation and enhances Nrf2 translocation to the nucleus

**Fig 5:**
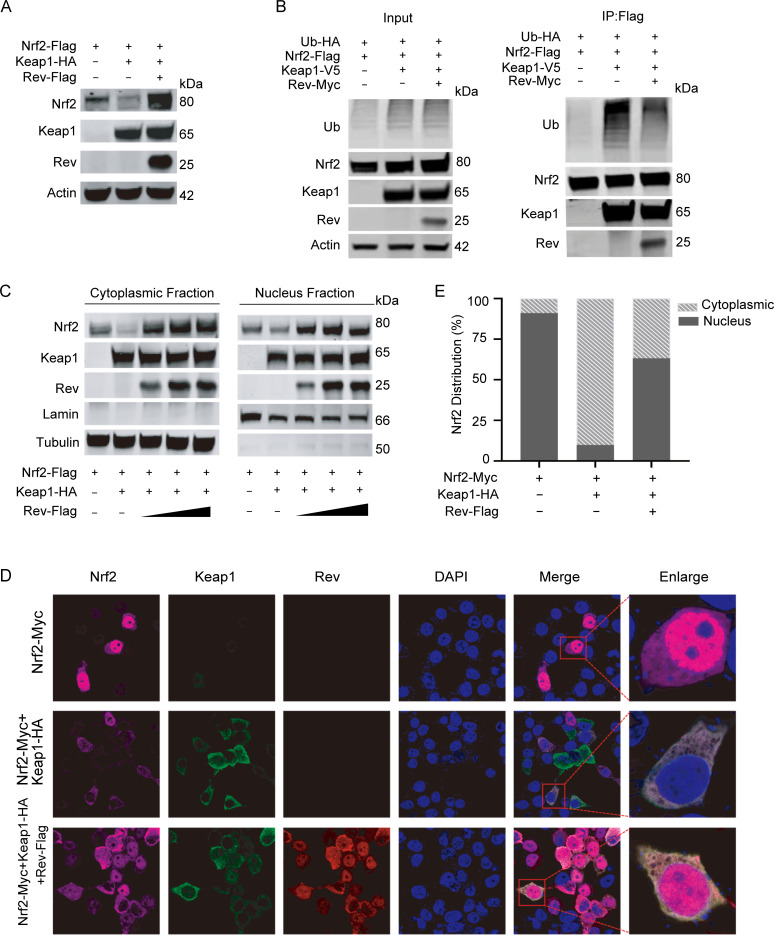
EAIV-Rev reduces Keap1-mediated Nrf2 ubiquitination and facilitates Nrf2 translocation into the nuclei. **(A)** Rev prevents Keap1-mediated Nrf2 degradation. Keap1 and *rev* were expressed in 293T cells alone or in combination as indicated. Cells were analyzed using western blotting at 24 h p.t. **(B)** Rev reduces Keap1-mediated Nrf2 ubiquitination. 293T cells were transfected with the indicated plasmids. At 24 h post-transfection, whole-cell lysates were immunoprecipitated using Flag beads and then ubiquitination was analyzed by blotting with HA-K48 ubiquitin antibody. **(C)** Protocol as in (A), with the addition of a step separating the cytoplasmic and nuclear proteins. Western blotting was performed as in Fig 3B. **(D)** The distribution of Nrf2 was visualized with or without Rev using confocal imaging. The plasmids encoding Nrf2 (purple) and Keap1 (green) were expressed alone or in combination with *rev* (red) in 293T cells. Twenty-four hours later, cells were fixed using acetone/methanol and subjected to immunofluorescence analysis using the indicated antibodies. Nuclei were visualized by staining with DAPI. Quantifications are given in **(E)**. In each transfection experiment, at least 100 cells were scored, and the gray and stripe bars represent cytosolic and nuclear localization, respectively. Each of these experiments was repeated at least twice, and consistent results were obtained.

We have shown that Rev binds to Keap1 and impairs the interaction between Keap1 and Nrf2. Given that Keap1 is known to mediate Nrf2 degradation via ubiquitination [35], we next tested whether Rev affects the status of Keap1-mediated Nrf2 degradation. First, Keap1 and Nrf2 were co-transfected into 293T cells with or without Rev. As expected, Rev was able to reverse Keap1-mediated degradation of Nrf2 and restore the levels of Nrf2 in the cytoplasm (Fig 5A, compare lanes 2 and 3). Moreover, overexpression of Rev resulted in a significant decrease in Keap1-mediated Nrf2 ubiquitination (Fig 5B). As EIAV infection did not impact Keap1 protein levels in host cells (Fig 1D and 1E), all these data indicated that the increase in Nrf2 was as a result of the reduction of Keap1-mediated Nrf2 degradation regulated by Rev. To provide further evidence of the ability of EIAV-Rev to release Nrf2 from its negative regulator Keap1, we investigated the distribution of Nrf2 with or without Rev co-expression with an immunofluorescence assay. When Nrf2 was ectopically expressed alone, it showed a nuclear distribution, whereas it exhibited a homogenous cytoplasmic distribution if co-expressed with Keap1 (Fig 5D). In cells co-expressing Nrf2 and Keap1, the addition of Rev resulted in translocation of Nrf2 from the cytoplasm to the nucleus (Fig 5E). These results further suggest that the interaction between Rev and Keap1 inhibits Keap1-mediated degradation of Nrf2, leading to the translocation of Nrf2 from the cytoplasm to the nucleus.

### Nrf2 inhibits EIAV virion production in equine macrophages and 293T cells

**Fig 6:**
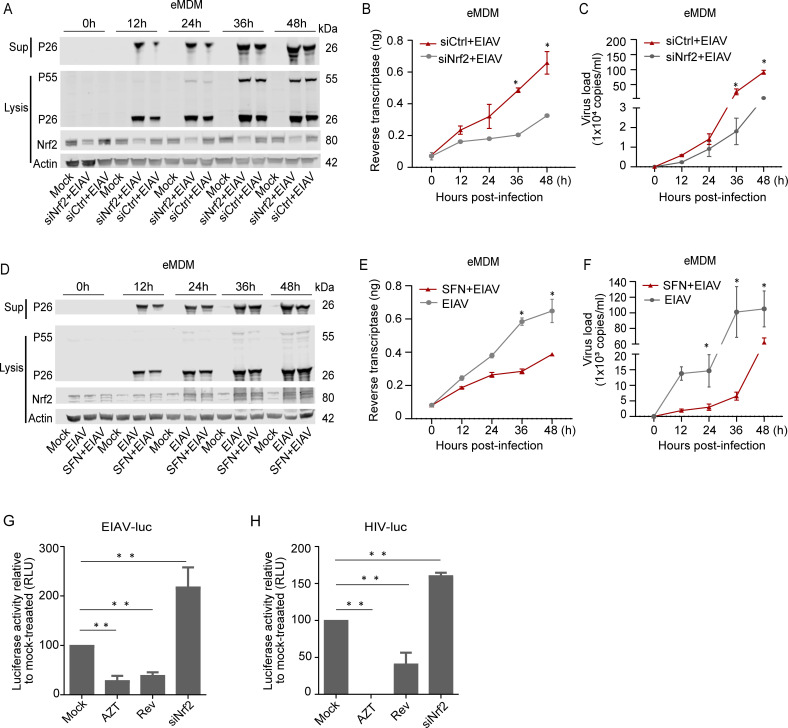
Nrf2 blocks EIAV replication in equine macrophages. **(A-C)** eMDMs were pre-treated with Nrf2-specific siRNA or scramble siRNA. Six-hours after treatment, cells were either mock infected or infected with EIAV at 1×10^5^ TCID50. 24 hours post infection, cell extracts were analyzed using western blotting. Viral replication was determined using real-time RT PCR and reverse transcriptase activity assays. **(D-F)** same procedure as in A-C, except that eMDMs were treated with media supplemented with 20 μM SFN. **(G-H)** 293T cells were transfected with *rev* or Nrf2-specific siRNA. After treatment for 12h, cells were inoculated with VSV-G-pseudotyped EIAV encoding firefly luciferase (K) and VSV-G-pseudotyped HIV-1 (L). Twenty-four hours later, luciferase activity was measured using photon emission. AZT (Zidovudine), a specific reverse transcription inhibitor, served as a positive control for viral inhibition. The data are expressed as means and SD for at least three independent replicates. *P <0.05, * *P<0.01.

So far, our data supported the idea that EIAV-Rev promotes Nrf2 function via competitively binding to Keap1. However, the role of Rev-mediated Nrf2 activation in EIAV production and replication remains unclear. To investigate this, we first knocked out the Nrf2 gene in 293T cells (Nrf2_ko_) and used an EIAV infectious molecular clone to transduce Nrf2_ko_ 293T cells. We found that the production of virions was significantly increased in cell lysate and supernatant of Nrf2_ko_ cells compared to the control 293T cells. Moreover, the increased virion production in Nrf2_ko_ cells was repressed when Nrf2 protein was re-constituted (S2A and S2B Fig). Furthermore, ectopic expression of Nrf2 in 293T cells repressed viral production in a dose-dependent manner (S2C and S2D Fig). Over expression of Keap1 could down regulate Nrf2 expression and enhance viral production (S2E and S2F Fig). These results indicated that Nrf2 plays an important role in combating EIAV replication. To further confirm this observation, we used equine macrophages, the natural host cells of EIAV, to verify the interaction between EIAV and the Nrf2 pathway. A specific siRNA interfering with Nrf2 expression and a specific Nrf2 activator (SFN) were used to pre-treat equine macrophages prior to EIAV infection. We then measured time-dependent expression of EIAV replication using real-time PCR and assays of EIAV reverse transcriptase activity (RT) in parallel. We observed that the EIAV-P26 protein levels both in cell lysis and in the cultural supernatant were gradually up-regulated in siNrf2 RNA-treated eMDMs compared to the siRNA-treated eMDMs (Fig 6A). Real-time PCR and RT activity analysis also showed time-dependent increases in the levels of virus replication (Fig 6B and 6C), indicating that knocking down Nrf2 significantly enhanced EIAV replication. In contrast, the activation of Nrf2 (SFN: 20 μM, 6h) reduced EIAV replication (Fig 6D–6F). Overall, these results indicate that Nrf2 is able to inhibit EIAV replication.

After we confirmed the ability of the Rev-triggered Nrf2 pathway to regulate EIAV infection, we next used a VSV-G pseudotyped *env* (–) EIAV luciferase reporter virus system to investigate whether Rev was able to serve as Nrf2-activator to suppress virus infection. The results showed that EIAV infection was obviously repressed with over-expression of Rev (Fig 6G). Most interestingly, the HIV-1 luciferase reporter system showed a similar response (Fig 6H). Together with the result from Figs 4 and 5, these data demonstrate that Keap1 can recognize and bind Rev to release Keap1, and further activate antiviral response to block EIAV and HIV-1 replication.

### Keap1 limited Rev-mediated viral mRNA export correlated with low virus production

**Fig 7:**
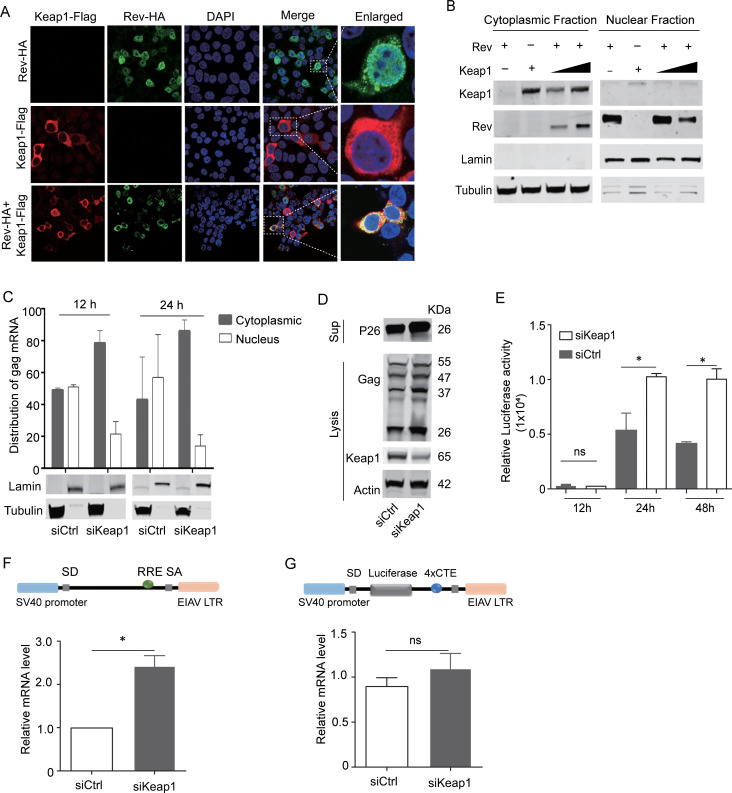
Keap1 hijacks *rev* in the cytoplasm and limits *rev*-mediated RNA transport. **(A)** Distribution of Rev was screened with or without Keap1 using confocal imaging. Scale bars 10μm. Images are representative of 3 independent experiments. **(B)** 293T cells were co-transfected with HA-tagged-*rev* and Keap1. The cytoplasmic and nuclear proteins were fractionated as in Fig 2B and then immunoblotted for Keap1 and Rev. **(C)** Distribution of cytoplasmic and nuclear unspliced *gag* RNAs in siKeap1 and control cells was analyzed using real-time PCR with primers specific for *gag* mRNA. Lamin and tubulin were used as nuclear and cytoplasmic controls, respectively. **(D)** 293T cells were treated with siRNA targeting Keap1 (siKeap1) or scrambled siRNA (siCtrl) and then transfected with EIAV_CMV3-8_. Cells were lysed and samples were analyzed for expression of Gag, Keap1, and Actin using western blotting. **(E)** EIAV pseudovirions were generated separately in siKeap1 or siCtrl cells and then used to infect 293T cells. The luciferase activity in the supernatant was assayed at the indicated timepoints (12 h, 24 h and 48 h). (**F)** Rev/RRE RNA export reporter plasmids were transfected into siKeap1 and control cells and viral mRNA synthesis was calculated using real-time RT PCR. **(G)** The protocol is as (F), but with the Rev-independent RNA export reporter plasmid (4×CTE). The graph represents three independent experiments; error bars represent results from SEM.

Having confirmed the reduced viral production resulting from Nrf2/Keap1 activation induced by Rev, we next asked whether the binding of Rev by Keap1 could also directly inhibit viral protein expression, as EIAV replication is known to be absolutely dependent upon Rev-mediated transport of viral RNA [36,37]. We first investigated the sub-cellular localization of Rev and Keap1. Our results suggested that Rev was located predominantly in the nucleus in the absence of Keap1, while in the presence of Keap1, Rev was located mostly in the cytoplasm and co-localized with Keap1 (Fig 7A and 7B). Subsequently, EIAV pseudotyped virus was used to infect siKeap1- or scrambled siRNA-treated cells. The nuclear and cytoplasmic RNA fraction were then isolated and purified, and the cytoplasmic and nuclear distributions of EIAV-*gag* mRNA were analyzed using quantitative PCR at 12 h and 24 h post infection. Our results showed that most of the *gag* mRNA had accumulated in the cytoplasm when Keap1 endogenous expression was silenced (Fig 7C). These data indicate that the efficiency of Rev-mediated RNA transport between the cytoplasm and nucleus is greatly enhanced in siKeap1 cells (Fig 7C). As a result of the increased transport of the primary *gag* transcript, viral production was higher in siKeap1 cells, compared with control cells under transfection and infection conditions (Fig 7D and 7E). These data indicate that Keap1 may limit Rev-mediated RNA transport through direct interaction with Rev. To investigate this phenomenon further, we compared the effects of Keap1 on RNA transported by different RNA export systems. We found that RNA transport was promoted in the Rev-dependent Rev/RRE export system (Rev-dependent) in the absence of Keap1. In contrast, there was no effect in the Rev-independent 4×CTE export system (Fig 7F and 7G). We therefore surmised that binding with Keap1 could lead to the accumulation of Rev in the cytoplasm and subsequently impair Rev/RRE dependent RNA transport, inhibiting *gag* mRNA production in trans and leading to reduced viral production. Taken together, these data indicate the dual roles of Keap1 in the antiviral response: 1) sensing the viral Rev protein to activate Nrf2-AREs pathway; 2) the direct binding and blocking of Rev to inhibit viral RNA transportation.

## Discussion

**Fig 8:**
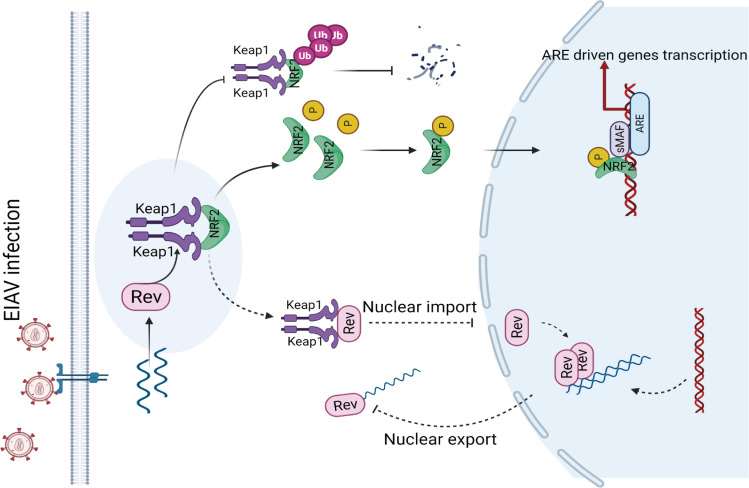
A proposed model for the cellular Nrf2/Keap1 axis manipulation of EIAV replication. Nrf2/Keap1 plays a crucial role in the management of oxidative stress. Under normal conditions, Keap1 interacts with Nrf2 in the cytoplasm, targeting it for proteasomal degradation to maintain redox homeostasis. Under EIAV infection, EIAV-Rev interacts with Keap1, disrupting the interaction of Keap1-Nrf2, leading to the enhancement of pNrf2 and nuclear translocation which results in the activation of Nrf2. The activation of the Nrf2 signal in turn inhibits EIAV replication via increasing expression of several antioxidant enzymes and also by limiting *rev*-mediated RNA transport.

Activation of the Nrf2/Keap1 axis through various mechanisms to regulate oxidative stress has been described from the infection of several viruses, including hepatitis C virus (HCV), HBV, influenza and HIV [31,38–40]. However, the mechanisms that underline Nrf2/Keap1 axis activation, especially the roles of Keap1 in antiviral defense, have not been fully elucidated. Here, we revealed a novel antiviral mechanism by which the Nrf2/Keap1 axis was activated following EIAV infection, and propose a working model of Keap1 (Fig 8). Briefly, Keap1, a specific repressor of Nrf2, can act as a sensor, directly binding EIAV-Rev upon EIAV infection and disrupting the binding of Keap1 and Nrf2, leading to the release and nuclear localization of Nrf2 and triggering the antioxidant response. At same time, as an effector, Keap1 binds and retains Rev in the cytoplasm, disrupts Rev’s function in the transportation of viral RNA, and further represses EIAV production in host cells.

Many viral infections can cause host cellular oxidative stress and further induce Nrf2 activation. Interestingly, some virus infections, such as that of Kaposi’s Sarcoma-Associated Herpes Virus (KSHV), benefit from Nrf2 activation [41–43]. However, Dengue virus and Respiratory syncytial virus (RSV) infection increase reactive oxygen species (ROS) levels and induce degradation of Nrf2 by different mechanisms [44–46]. As the key molecule that regulates the Nrf2-ARE pathway, Keap1 binds to Nrf2 to maintain a cellular antioxidant defense homeostasis. Little is known of the role of Keap1 in the regulation of viral infection except that Marburg virus (MARV) VP24 targets Keap1 to drive activation of Nrf2, which is likely to contribute to viral infection [30,47]. Previous research has demonstrated that HIV-1 Tat protein can activate the Nrf2 pathway, which in turn suppress Tat-induced HIV-1 LTR transactivation [48]. Here we report novel roles for Keap1 in the defense against equine lentivirus EIAV infection through directly targeting the viral accessory protein Rev. That the direct binding of Rev by Keap1 inhibits viral RNA transportation is unique and has not been observed in other viruses. This evidence suggests that the cellular Keap1/Nrf2 pathway is able to utilize virus-coded protein as a trigger to exert anti-viral defenses.

The Keap1-Nrf2 interaction is required for negative regulation of the anti-oxidant pathway [49]. We found that EIAV Rev binds to the Kelch domain of Keap1, which is the same domain used to bind Nrf2. Because Keap1-Rev and Keap1-Nrf2 have similar binding affinities and dynamics, Keap1 recognition of and interaction with Rev results in release of Nrf2 from Keap1, with subsequent Nrf2 accumulation in the nucleus and increase in Nrf2-dependent ARE activity. Two types of Nrf2/Keap1 activation, the “canonical” and “non-canonical” mechanisms, have been documented [49,50]. Oxidative or electrophilic compounds cause electrophilic-modification of one or more cysteines in Keap1, which results in a conformational change that reduces the ability of Keap1 to bind with Nrf2 [49,50]. This is known as the “canonical” activation pattern [49]. Here, we found that EIAV Rev could disrupt the Keap1-Nrf2 interaction through direct interaction with Keap1. Hence, the Nrf2 activation induced by EIAV Rev belongs to the “non-canonical” pattern, through disruption of the Keap1-Nrf2 complex via interaction with Keap1 or Nrf2 [49,50]. Several cellular factors, including P62, BRC interaction A1 and DPP3, are known to be able to reduce or abolish the binding of Keap1 with Nrf2 in this way [49]. However, with the exception of the Marburg virus VP24 [47], virus-coded proteins have only rarely been reported to be involved in the regulation of the Nrf2/Keap1 axis act through direct interaction.

Keap1 interactors including Nrf2, P62, WTX, and prothymosin alpha interact with Keap1 arginine residues mainly through electrostatic interactions [51–53]. Keap1 interactors also displayed acidic residues along with a “GE motif” [54,55]. It has been confirmed that Keap1 binding to Nrf2 occurs via two recognition sites (the “ETGE” and “DLG” motifs), forming a “hinge-latch” like structure [55]. Keap1-mediated repression of Nrf2 is dependent on this structure [54]. Given that a “GE motif”-like sequence was found at the Rev C-terminal, we speculated that the interaction of Keap1 and Rev has a similar mechanism as the Keap1-Nrf2 interaction. However, the mutation of these two motifs to alanine was not sufficient to reduce or abrogate the binding of Rev and Keap1. However, the N terminal (1-56aa) of Rev was found to be necessary for its binding with Keap1, as well as Keap1-dependent Nrf2 activation, without exhibiting any similarity with “GE motifs”. These discoveries suggest that Rev might present other motifs for its interaction with Keap1. It has been reported that “non-covalent” Nrf2 activators could be potential candidates to break up the Keap1-Nrf2 complex due to their specificity or the ability to avoid prolonged Nrf2 activation [50,56,57]. Because of this, some peptides derived from the “ETGE” motif of Nrf2 or some other small molecules have the ability to disrupt the Keap1-Nrf2 complex, making Nrf2-targeted therapeutics possible [22,27,57]. Therefore, uncovering the residues responsible for the interaction between Rev and Keap1 will also benefit exploration for novel drugs for Nrf2-targeted therapeutics.

Taken together, the findings reported here provide insights into the molecular mechanism underlying viral infection induced Nrf2/Keap1 axis activation and reveal a novel paradigm for how cellular pathways can target virus components to facilitate anti-virus defense functions. Additionally, our results identify potential domain sequences that are responsible for Keap1 binding and triggering the Nrf2/Keap1 axis to control EIAV replication, and perhaps also that of HIV-1. The current studies highlight the significance of the Nrf2/Keap1 axis in the regulation of EIAV replication and broaden our understanding of antioxidant-stress induced by virus infection.

## Materials and methods

### Reagents and antibodies

Sulforaphane (SFN) (S8044) and Zidovudine (AZT) (L0350) were bought from LKT Laboratories. The mouse anti-actin (A1978), mouse anti-Flag (F1804), and mouse anti-HA (H9658) monoclonal antibodies; the rabbit anti-Flag (F7425), rabbit anti-HA (H6908), and anti-Mouse IgG-FITC antibodies were purchased from Sigma. Rat anti-Myc (ab206486), mouse anti-Tubulin (TA503129) monoclonal antibodies, Rabbit anti-Lamin (EPR8985), Alexa Fluor 568-conjugated (ab175476) and Alexa Fluor 647-conjugated (ab190565) antibodies were purchased from Abcam (Cambridge, UK). The rabbit anti-GST (10000-0-AP) antibodies were purchased from Proteintech. DyLight 800-labeled goat anti-mouse (5230–0415) and DyLight 508 680-labeled goat anti-rabbit (5230–0403) secondary antibodies were purchased from KPL. Anti-eqNrf2, anti-eqKeap1 and anti-P26 antibodies were prepared in our laboratory. The nuclear extraction kit was purchased from Invent Biotechnologies (SC-003). Non-cytotoxic dosages were used in this study.

### Plasmid construction, transfection and infection

The eqNrf2 and eqKeap1 cDNA used in this study were cloned from equine monocyte-derived macrophages (eMDMs) using RT-PCR with the following set of primers:

eqNrf2 sense, 5’- ATGATGGACTTGGAGGTGC -3’;

eqNrf2 anti-sense, 5’- CTTCTTCTTGACGTCTGTCTTC-3’;

eqKeap1 sense, 5’- ATGTTCGCGTCCACCGAGTGCAAGG-3’;

eqKeap1 anti-sense, 5’- ACAGGTACAGTTCTGCTGGTC-3’.

The eqNrf2 constructs were obtained by cloning PCR-generated fragments into the VR1012 vector tagged with Flag or Myc at the N-terminal. The eqKeap1 expression vector was inserted into a pcDNA3.1 expression vector with an HA tag at the C-terminal. All expression vectors were generated using the In-Fusion cloning (Clontech, Felicia, CA). A series of deletion mutants of eqkeap1 (eqKeap1-ΔNTR, eqKeap1-ΔBTB, eqKeap1-ΔIVR, eqKeap1-Δkelch, eqKeap1-ΔCTR-HA) were generated using standard oligo-directed mutagenesis techniques. Constructs of *rev* and its mutants were created using the same techniques. For transfections, the corresponding plasmids were transfected using Poly Jet transfection reagent (SignaGen Laboratories, SL100688) after the 293T cells had been grown to 80% confluence for 18–24 h before treatments. Cell lysates and culture supernatants were collected 24 hours after transfection. For infection, the equine monocytes were infected with EIAV at 1×10^5^ TCID50. Cell extracts were collected and analyzed according to the requirements of the experiment.

### Microarray analysis

Equine macrophages were infected at an MOI of 10 as described above, cells were collected at the indicated times and TRIzol reagent (Qiagen) was used for RNA extraction, according to the manufacturer’s protocol. RNA purity was evaluated and RNA quantified using a NanoDrop 2000 spectrophotometer (Thermo Scientific, USA). RNA integrity was assessed using an Agilent 2100 Bioanalyzer (Agilent Technologies, Santa Clara, CA, USA). Libraries were then constructed using TruSeq Stranded mRNA LT Sample Prep Kit (Illumina, San Diego, CA, USA) according to the manufacturer’s instructions. Transcriptome sequencing and analysis were performed by OE Biotech Co., Ltd. (Shanghai, China). The libraries were sequenced on an Illumina HiSeq X Ten platform and 150 bp paired-end reads were generated. About 59437095 raw reads for each sample were generated. Raw data (raw reads) of fastq format were first processed using Trimmomatic [58] and the low quality reads were removed to obtain clean reads. About 58897197 clean reads for each sample were retained for subsequent analyses. The clean reads were mapped to the human genome (GRCh38) using HISAT2. FPKM of each gene was calculated using Cufflinks, and the read counts for each gene were obtained by HTSeq-count [59]. Differential expression analysis was performed using the DESeq (2012) package in R [60]. A p value < 0.05 and fold change > 2 or fold change < 0.5 was set as the threshold for significantly differential expression. Hierarchical cluster analysis of differentially expressed genes (DEGs) was performed to demonstrate the expression patterns of genes in different groups and samples. GO enrichment and KEGG pathway enrichment analyses of DEGs were performed separately using R, based on the hypergeometric distribution. The pathway enrichment and network analyses were performed based on this calculation: ratio of the number of up-regulated genes to the total number of genes that map to the canonical pathway.

### Activation of the Nrf2/Keap1 axis

For ARE reporter gene assays, 293T cells were co-transfected with the plasmids expressing ARE-inducible firefly luciferase (pARE-Luc; Beyotime) and the following plasmids expressing viral proteins: VR1012-*gag* /pcDNA3.1-*env* /pcDNA3.1-*S2* /pcDNA3.1-*Tat* /VR1012-*rev* separately, using the Poly Jet transfection reagent as previously described. After 24 h, a dual luciferase reporter assay (Promega, E1500) was performed in triplicate and firefly luciferase values were normalized to *Renilla* luciferase values. The expression of the corresponding target proteins was detected using western blotting. Levels of endogenous mRNAs from Nrf2 and Nrf2-downstream genes (NQO1, OAS1 and HMOX1) were assessed using quantitative real-time RT-PCR with 2×SYBR Green Fast qRT-PCR Master Mix (Biotool). The value obtained for each gene was normalized to β-actin expression. The sequences of primers used in this study were as follows:

eqNrf2 sense: 5’-ATGGATTTGATTGACAT-3’;

eqNrf2 anti-sense: 5’-TCACCTGTCTTCATCTAGTT-3’;

eqNQO1 sense: 5’-TGGAAGGATGGAAGAAACG-3’;

eqNQO1 anti-sense: 5’-GGACTTGCCCAAGTGATG-3’;

eqOAS1 sense: 5’-AGGCTACCCCAATATGAATCA-3’;

eqOAS1 anti-sense: 5’-ATCCCCAAGGCCCATC-3’;

eqHMOX1 sense: 5’-CCCAGGATTTGTCAGAGGCC-3’;

eqHMOX1 anti-sense: 5’-TGGTACAGGGAGGCCATCAC-3’.

### Immunoprecipitation, immunoblot analysis, and ubiquitination assay

These experiments were performed as previously described. For immunoprecipitation, samples were prepared after transfection with the indicated plasmids, and then incubated overnight with the appropriate antibodies together with anti-HA magnetic beads (Sigma-Aldrich, A2095), anti-Flag magnetic beads (Sigma-Aldrich, A2095) or anti-GST magnetic beads (Genscript, L00327). The beads were washed three to five times with ice-cold PBS and incubated with the cell lysates overnight at 4°C. The immunoprecipitants were washed three times in 1 ml SDS lysis buffer and subjected to immunoblot analysis. For *in vitro* ubiquitination experiments, 293T cells were transfected with the indicated plasmids for 48 h. Cells were lysed under denaturing conditions in an SDS buffer (50 mM Tris-HCl, pH 7.5, 0.5 mM EDTA, 1 mM DTT, 1% SDS) by boiling for 10 min. The lysate was subjected to immunoprecipitation using anti-Flag magnetic beads and subsequent SDS-PAGE. Ubiquitylated Nrf2 was detected with anti-HA antibody. The protein band was detected using a Licor Odyssey imaging system (USA) and quantified using ImageJ software.

### Proximity ligation assay (PLA)

A proximity ligation assay (PLA) was performed to detect protein-protein interactions using fluorescence microscopy as previously described [61,62]. Briefly, 293T cells were cultured in 10-chamber microscopic slides, and then co-transfected with *rev* and Keap1 or *rev* and Nrf2. 18 h later, the cells were fixed with 4% paraformaldehyde for 15 min, and blocked with DuoLink blocking buffer for 1 h at 37°C. Cells were then incubated with rabbit anti-HA and mouse anti-Flag monoclonal antibodies diluted with the specific DuoLink antibody diluents for 2 h, washed for 2 minutes in 1× wash buffer A, and further incubated for 1 h at 37°C with specific PLA probes under hybridization conditions. A ligase was then added to the cells for 30 min at 37°C, to form a concatemeric product extending from the oligonucleotide arm of the PLA probe. The PLA dot was visualized as distinctly fluorescent in the Texas red channel.

### Confocal microscopy

293T cells were plated in glass coverslips on 35-mm-diameter plates. Cells were co-transfected with Nrf2 and Keap1 expression plasmids either with or without *rev*. 24 h after transfection, cells were fixed with 4% paraformaldehyde (Beyotime) for 30 min, and rinsed three times with cold phosphate-buffered saline (PBS). The fixed cells were then permeabilized for 10 min with 0.1% Triton X-100 and blocked in 5% fat-free milk in PBS for 1 h, then incubated with anti-HA, Myc or Flag tags for 1 h. Cells were washed three times with cold PBS. Then anti-mouse primary antibody, conjugated with fluorescein isothiocyanate (FITC) or Alexa Fluor 568 or Alexa Fluor 647, was added to the coverslips and the whole was incubated for 1h. Nuclei were labeled with 4′, 6-diamidino-2-phenylindole (DAPI) (Beyotime, P0131). All antibodies for immunostaining here were used at 1:500 dilution. The images were captured using a confocal microscope (LSM 880; Zeiss, Germany).

### CRISPR-Cas9 and RNAi

The Nrf2 knockout cell line was generated using CRISPR-Cas9 technology. The CRISPR-Cas9 lentiviral vector lentiCRISPR v2-cas9-GFP was purchased from Addgene (USA). The gRNA sequence used for targeting Nrf2 is GCGACGGAAAGAGTATGAGC. CRISPR/Cas9-mediated mutagenesis of Nrf2 was conducted following a previously described protocol [63]. 24 h after transfection, limiting dilution was used to allocate a single GFP-positive cell per well in 96-well plates using flow sorting. The recovered KO clones were validated using DNA sequencing and western blotting.

The Keap1 target-specific siRNAs were designed according to the Keap1 sequence and synthesized by Sigma. The nonspecific siRNA was used as a negative control to confirm the specificity of the inhibition. The 293T cells were transfected with a pool of 3 Nrf2-targeting siRNAs (TGACAGAAATTGACAACAA; AGACAAGAACAACTCCAAA; ACAGTGTCTTAACATTCAA) or control siRNA (CAAACAGAAUGGUCCUAAA) (50mM) using Lipofectamine RNAiMAX transfection reagent (Invitrogen, 13778100) according to the manufacturer’s instructions. The efficiency of silencing of the target gene was evaluated using western blotting.

### Protein expression and purification

cDNA encoding full-length equine Nrf2 (Genbank No. XM_008536608.1) and Keap1 (Genbank No. XM_023645423.1) were PCR-amplified from equine macrophage cells and subcloned into a pET vector containing C-terminal His6, and a PEGX6P vector with C-terminal GST tag separately. cDNA encoding EIAV *rev* was subcloned into a modified PET29a with a C-terminal MBP. For protein expression, BL21 (DE3) competent or Rosetta (DE3) competent *Escherichia coli* cells were used. Protein expression in *E*. *coli* cells was induced with 0.3 mM isopropyl-D-thiogalactopyranoside (IPTG) overnight at 18°C. His-tagged Nrf2 protein and MBP-tagged Rev protein were purified with nickel affinity (HisTrap; GE Healthcare), eluted with imidazole, and desalted into binding buffer. GST-tagged Keap1 was purified on glutathione-Sepharose 4B (GE Healthcare), eluted with 10 mM reduced glutathione in 50 mM Tris, pH 8.0, and 150 mM NaCl. Protein desalinations were further performed using Akta Avant (GE Healthcare). Purity of the proteins was monitored at all stages of the purification process, and proteins were visualized using Coomassie blue staining.

### Bio-layer interferometry assay (BLI)

Binding assays were performed using an Octet Red instrument (ForteBio) based on biolayer interferometry. Streptavidin-coated biosensors were loaded with biotinylated-Keap1 in PBST (phosphate-buffered saline with 0.1 mg/ml BSA, 0.002% Tween 20, pH 7.2) at a concentration of 65 μg/ml. The loaded biosensors were washed in the same buffer and then incubated together with various concentrations of Rev or Nrf2 in PBST for the indicated times to allow association. To achieve binding competition affinities, we engineered the biotinylated-Keap1 onto the streptavidin-coated biosensor first for 600 s, washed it in buffer for 180 s, and incubated it with the optimal concentrations (calculated from the affinities of Rev and Keap1) of Rev or buffer in PBST for 60 s to allow association. It was then incubated together with Nrf2. Sensors incubated together with buffer instead of Rev formed the control. The reverse competitive binding experiment was conducted in the same format. Assays were performed at room temperature in black 96-well plates (E&K Scientific). Kinetic parameters (kon and koff) and affinities (KD) were calculated using the Octet software v.6.1.

### Production of VSV-G pseudotyped retroviruses

Briefly, for rescue of the EIAV pseudovirions, 293T cells were seeded in 100-mm dishes and transfected with pONY8.1-Luc, pEIAV-GagPol and vesicular stomatitis virus glycoprotein (VSV-G) expression plasmids using the PEI transfection reagent (Polysciences) as described previously. The culture supernatants were collected at 48 h post-transfection and were then centrifuged at 20,000 rpm at 4°C for 2 h. Viral pellets were resuspended in lysis buffer and subjected to western blotting analysis.

### Construction of equine arteritis virus (EAV) with Rev

An equine arteritis virus infectious clone expressing eGFP between nsp1 and nsp2 gene (EAV-eGFP) was established by Qi. (2017). In this study, EIAV-*rev* was inserted into the EAV genome by replacing the eGFP gene of EAV-eGFP. Briefly, the whole EAV infectious clone except the eGFP gene was amplified from the plasmid EAV-eGFP with the following primers: EAV sense: 5’-GCGCCTGTGAAACAGCTTCTGAAC-3’ and EAV antisense: 5’-TGGTGGATTGTAGCCGCCGTAGTTG-3’. *rev* carrying a Flag tag at the C terminal (*rev*-Flag) was amplified with the following primers: *rev*-Flag F: 5’-ACGGCGGCTACAATCCACCAATGGCAGAGGCAAGAGACACAAG-3’ and *rev*-Flag R: 5’-AGAAGCTGTTTCACAGGCGCCTTATCATCATCGTCCTTATAATCTAGATGTTTCCTCCTTCGCTTTG-3’. Finally, the EAV infectious clone expressing Rev-Flag was generated using the In-Fusion cloning.

### Cell fraction and quantitative real-time PCR

293T cells were pre-treated with Keap1 target-specific siRNA or control siRNA and then infected with EIAV pseudotyped virus. Cells were collected at 12 h and 24 h post infection. To quantify the mRNA in siKeap1 and siCtrl cells, total RNA from the cells was extracted using a Bio-fast simply RNA extraction kit (catalog # BSC60S1, Bioer). Nuclear and cytoplasmic RNA fractions were isolated and purified using the PARIS Kit (catalog # AM1921, Thermo Scientific) according to the manufacturer’s instructions. An equal volume of RNA was used for cDNA synthesis using the High-Capacity cDNA reverse transcription kit (catalog # R223-01, Vazyme Biotech). Real-time PCR was then performed using the SYBR green PCR mixture with specific primers targeting *gag*. A relative quantification method was used, with Lamin and Tubulin protein as the nuclear and cytoplasmic protein controls, respectively. The following primers were used: Gag sense, 5’-GGGATTATTTGGTAAAGGG-3’, Gag anti-sense, 5’-GATTCTGCCATGCTGTTCT-3’.

### Quantification of viral replication and growth

We quantified EIAV replication and growth using RT-qPCR and reverse transcriptase activity (RT) separably as previously described [7.11]. For RT-qPCR, reactions mixes included reverse transcribed cDNA, 1 × TaqPath 2-Step RT-qPCR Master Mix (Takara), forward and reverse primers, and the probe. The primers and probe used in the process were: Gag sense, 5’-CGATGCCAAATCCTCCATTAG-3’; Gag anti-sense, 5’-CTGATCAAAAGCAGGTTCCATCT-3’; Probe:5’-FAM-CACCACAAGGGCCTATTC CCATGACA-TAMRA-3’. Viral copy number was assessed using Gag qPCR standards at 10 X dilution to generate standard curves. Viral growth was quantified in parallel using reverse transcriptase activity assays as described previously [7.11].

### Statistics

All the graphs presented in this study were created in GraphPad Prism version 6.0 (La Jolla, CA, USA). The statistical values were calculated using one-way ANOVA or two-tailed Student’s *t*-tests. Each data bar represents the mean value ± SD (standard deviation) of at least three independent experiments in all cases. Asterisks indicate the statistical significance: ns, no significance; *, P < 0.05; **, P < 0.01.

## Supporting information

S1 Fig The N-terminal of EIAV-*rev* is necessary for Keap1-Rev interaction and Rev-triggered Nrf2 activation. **(A)** Schematic diagrams showing the mutations of *rev* used in B. **(B)** Western blot was performed to evaluate the interactions between Keap1 and *rev* mutants. 293T cells were transfected with expression plasmids carrying the indicated VR1012-based *rev* and its mutants with Keap1 and then analyzed with Co-IP using anti-Flag antibody. **(C)** Schematic diagram of WT EIAV-*rev* and its truncations used in D and E. **(D)** The assay protocol is as (B) but cells were transfected with *rev*-Δ1–56, *rev*-Δ57–116 or *rev*-Δ117–165 mutants. **(E)** The ARE gene reporter was used to evaluate the Nrf2/Keap1 axis activation triggered by *rev* mutants.
(TIF)

S2 Fig Nrf2 inhibits EIAV replication in 293T cells. **(A-B)** Viral replication was determined in 293T and Nrf2_ko_ 293T cells using western blotting (A) and reverse transcriptase activity assays (B). **(C-D)** Viral replication was evaluated in 293T cells co-transfected with EIAV_CMV3-8_ and different amounts of plasmids expressing Nrf2. Viral protein expression in virions (C) and supernatant (D) was calculated as described in A and B. **(E-G)** 293T cells were transfected with EIAV_CMV3-8_, EIAV_CMV3-8_ plus Nrf2 with or without Keap1 plasmids. Cell lysates and supernatants were analyzed as in (C-D).
(TIF)
