# Supplementary figures and images for: Keap1 recognizes EIAV early accessory protein Rev to promote antiviral defense
Source: PLoS Pathog. 2022 Feb 9;18(2):e1009986. doi: 10.1371/journal.ppat.1009986 (PMC8863222; doi:10.1371/journal.ppat.1009986)

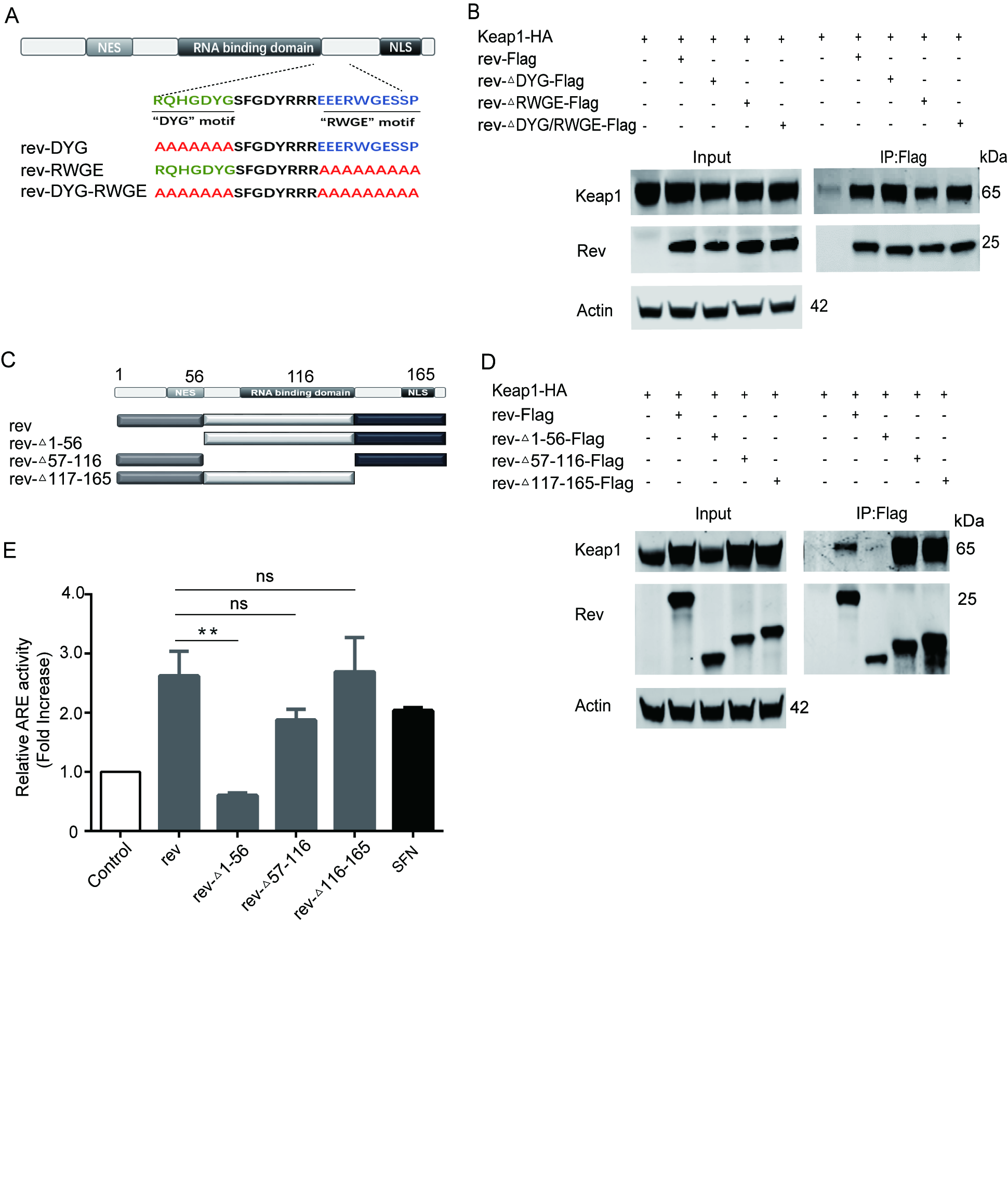

Supplement: S1 Fig — (A) Schematic diagrams showing the mutations of rev used in B. (B) Western blot was performed to evaluate the interactions between Keap1 and rev mutants. 293T cells were transfected with expression plasmids carrying the indicated VR1012-based rev and its mutants with Keap1 and then analyzed with Co-IP using anti-Flag antibody. (C) Schematic diagram of WT EIAV-rev and its truncations used in D and E. (D) The assay protocol is as (B) but cells were transfected with rev-Δ1–56, rev-Δ57–116 or rev-Δ117–165 mutants. (E) The ARE gene reporter was used to evaluate the Nrf2/Keap1 axis activation triggered by rev mutants. (TIF) [file ppat.1009986.s001.tif]

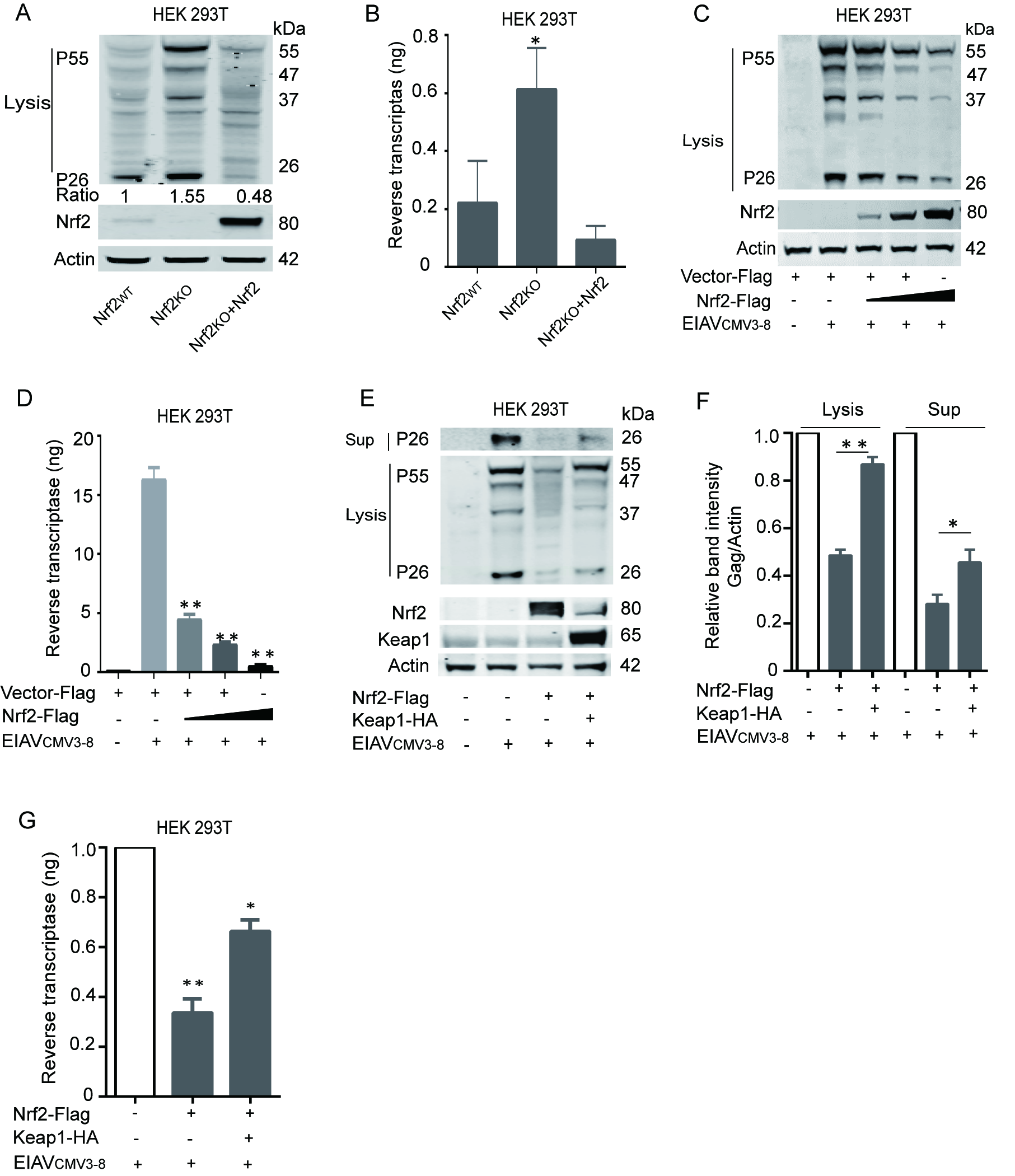

Supplement: S2 Fig — (A-B) Viral replication was determined in 293T and Nrf2ko 293T cells using western blotting (A) and reverse transcriptase activity assays (B). (C-D) Viral replication was evaluated in 293T cells co-transfected with EIAVCMV3-8 and different amounts of plasmids expressing Nrf2. Viral protein expression in virions (C) and supernatant (D) was calculated as described in A and B. (E-G) 293T cells were transfected with EIAVCMV3-8, EIAVCMV3-8 plus Nrf2 with or without Keap1 plasmids. Cell lysates and supernatants were analyzed as in (C-D). (TIF) [file ppat.1009986.s002.tif]
